# Combinational therapy of all-trans retinoic acid (ATRA) and sphingomyelin induces apoptosis and cell cycle arrest in B16F10 melanoma cancer cells

**DOI:** 10.55730/1300-0152.2715

**Published:** 2024-10-14

**Authors:** Zeynep İŞLEK KÖKLÜ, Elif Lidya ŞANVERDİ, Başak KARADAĞ, Mehmet Hikmet ÜÇIŞIK, Ezgi TAŞKAN, Fikrettin ŞAHİN

**Affiliations:** Department of Genetics and Bioengineering, Faculty of Engineering, Yeditepe University, İstanbul, Turkiye

**Keywords:** All-trans retinoic acid, sphingomyelin, melanoma, combinational chemotherapy, apoptosis, cell cycle arrest

## Abstract

**Background/aim:**

Melanoma arises from the uncontrolled multiplication of melanocytes, and poses an escalating global health concern. Despite the importance of early detection and surgical removal for effective treatment, metastatic melanoma poses treatment challenges, with limited options. Among optional therapies, including chemotherapy and immunotherapy, all-trans retinoic acid (ATRA), a natural metabolite of vitamin A, has shown promise in treating melanoma by inducing differentiation, apoptosis, growth arrest, and immune modulation in melanoma cells. However, ATRA treatment alone can lead to resistance and relapse. Furthermore, sphingomyelin (SM) was implicated in the inhibition of cell proliferation, differentiation, and apoptotic cell death during melanoma progression.

**Materials and methods:**

The combinational anticancer effects of ATRA and SM on an in vitro B16F10 melanoma model were investigated based on cell viability, apoptotic cell death, cell cycle progression, and gene expression levels; whereas the safety properties of the treatments were tested on RAW264.7 macrophages.

**Results:**

The combination of 123 μM of ATRA + 136 μM of SM was the most effective treatment, showing a 50% reduction in cell proliferation, leading to 53.91% apoptotic cell death in 48 h, and G2/M phase-cell cycle arrest in the B16F10 cells. While 123 μM of ATRA alone did not change the caspase 3 and Bax gene expressions, the combinational ATRA + SM treatment resulted in 2- and 5-fold increases in the gene expression level, respectively. A 13-fold increase in cyclin-dependent kinase inhibitor 2A was observed with the combinational ATRA + SM treatment, while suppressing the programmed death ligand 1 (PD-L1) expression by 0.5-fold.

**Conclusion:**

Combinational ATRA and SM therapy could be a promising therapeutic approach for melanoma, potentially improving efficacy, while reducing toxicity to healthy cells.

## Graphical Abstract

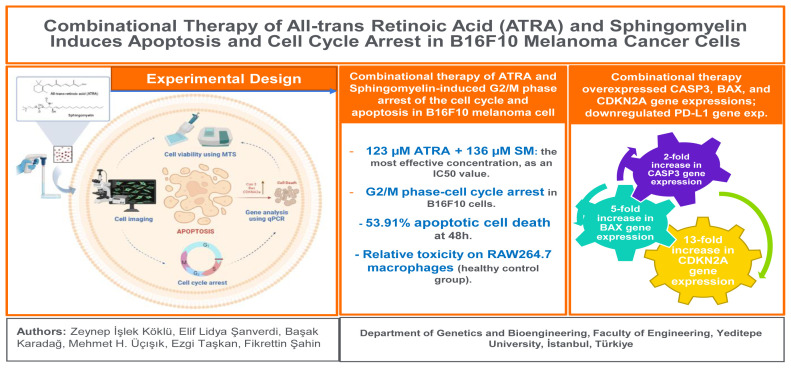

The figure was created with Biorender.com.

## 1. Introduction

The uncontrolled multiplication of melanocytes, skin pigment-producing cells, is the cause of melanoma, a type of malignant skin cancer. Despite being generally known to develop on the skin, melanoma can also occur on mucosal surfaces inside the body or in other regions including the uveal tract, where neural crest cells migrate^1^. Skin cancer is the prominent cause of malignancy and invasive melanoma accounts for around 1% of all skin malignancies; however, it is responsible for the majority of mortalities^1^. An estimated 325,000 new cases of melanoma (174,000 males and 151,000 females) were identified worldwide in 2020, with 57,000 deaths (Arnold et al., 2022).

While early detection and surgical removal of tumors remain the cornerstone of therapy, metastatic melanoma remains difficult to treat with limited treatment options. Therapy strategies, such as chemotherapy, targeted therapy, radiation therapy, surgical operation, and immunotherapy vary depending on the stage of melanoma, the thickness and growth rate of the tumor, and the genetic type of the melanoma (Garbe et al., 2011). The most effective treatments are immunotherapy and radiotherapy compared to chemotherapy with the Food and Drug Administration-approved drug for melanoma, i.e. dacarbazine, which is less frequently preferred (Lens and Eisen, 2003). Despite its potent action, the therapeutic impact of dacarbazine in the treatment of melanoma cancers is diminished by its poor water solubility and brief half-life (41 min) in blood circulation (Kakumanu et al., 2011). Moreover, the limitations of a single agent therapy were demonstrated by a depressing response rate of between 10% and 25% and less than 5% total cancer recovery (Ding et al., 2011; Almousallam et al., 2015). Therefore, there is a high demand for novel chemotherapeutic approaches in the treatment of melanoma. Recent studies have provided evidence of the promising biological activities of all-trans retinoic acid (ATRA), and sphingomyelin (SM) on melanoma (Jenkins et al., 2011; Espaillat et al., 2015).

ATRA, a natural metabolite of vitamin A, has shown therapeutic potential against melanoma by inducing differentiation, apoptosis, growth arrest, and immune modulation in cancer cells (Giuli et al., 2020; Liang et al., 2021; Jin et al., 2022). Specifically, ATRA mediates the transcription of many cell differentiation genes on retinoic acid receptors (RARs) in a variety of cancers such as acute promyelocytic leukemia and melanoma, resulting in the activation of ATRA-dependent RARs and retinoid X receptors in the nucleus, thereby providing differentiation in the cancer cells (Bahmad et al., 2019; De et al., 2020). ATRA treatment also leads to a decrease in DNA synthesis, alterations in cellular morphology, and extended doubling time, and the induction of cell cycle arrest, specifically in the G1 phase through the suppression of oncogenic signaling pathways (Brown, 2023). The current preclinical and clinical evidence provides support for its utilization in the context of melanoma treatment, which was approved under the name Vesanoid for the treatment of acute promyelocytic leukemia that does not respond to any chemotherapy (Čolović et al., 1997). While ATRA has demonstrated significant efficacy in treating several forms of cancer such as melanoma, it is important to note that the use of ATRA as a standalone treatment can result in the development of resistance and subsequent relapse (Özpolat, 2009). Therefore, new strategies can offer valuable insights into the development of the advancement of strategies focused on enhancing the administration of ATRA as a standalone therapeutic agent or in combination with other treatments, as well as overcoming drug resistance.

In addition, ATRA has an impact on the immune system through the differentiation of myeloid-derived suppressor cells (MDSCs) into macrophage and dendritic cells at the level of preclinical and clinical phase studies. In two clinical studies, ATRA was targeted in combination with immunotherapeutics to MDSCs in patients with cancer, including metastatic renal and lung cancers (Tobin et al., 2018; Joshi and Durden, 2019). Two clinical studies demonstrated that decreasing the quantity of MDSCs leads to improved patient survival. A phase 2 clinical study investigated the efficacy of combining ATRA with ipilimumab in individuals diagnosed with stage 4 melanoma (Joshi and Durden, 2019). Furthermore, ongoing clinical trials are investigating the efficacy of combining the programmed death ligand 1 (PD-L1) inhibitor pembrolizumab (Keytruda) with ATRA (Vesanoid) as a treatment for metastatic melanoma (Tobin et al., 2023). The clinical research assess the efficacy of the combination by decreasing the quantity and inhibitory function of immunosuppressive MDSCs in the peripheral blood of melanoma patients, hence halting the advancement of the illness. Considering the chemotherapeutic approach together with immunotherapy, the combinational treatment of ATRA and sphingolipids have been identified as potential anticancer agents that exhibit synergistic effects by inducing apoptosis and inhibiting cancer cell proliferation (Espaillat et al., 2015).

SM, a specific class of sphingolipids found in cell membranes, has lately gained attention in cancer research owing to its potential anticancer effects as tumor-suppressing biologically active lipids (Feng et al., 2014; Ogretmen, 2017; Li et al., 2021; Petrusca et al., 2022). The modulation of numerous signaling pathways involved in melanoma progression, such as the mitogen-activated protein kinase and phosphoinositide 3-kinase/protein kinase B (AKT) pathways, has been engaged in the regulation of cell proliferation, differentiation, and apoptotic cell death in melanoma cells (Sattar et al., 2021; Lee et al., 2023). Moreover, SM has been reported to inhibit the growth and spread of melanoma cells by inducing cell death and limiting the formation of new blood vessels in the tumor (Sheridan and Ogretmen, 2021). The anticancer properties of SM have been linked with the accumulation of ceramide and induction of apoptosis (Saddoughi et al., 2008; Oskouian and Saba, 2010). Over the SM cycle, the destruction of SMs and the production of phosphocholine and ceramide result in the activation of neutral sphingomyelinases (nSMase). Ceramides reduce protein phosphorylation, and expression of protooncogenes such as c-Myc, and modulate apoptosis (Oskouian and Saba, 2010; Ito et al., 2012 ). León et al. (2006) showed that C2-ceramide has a cytotoxic effect on the SK-MEL-1 melanoma cell line, which was consistent with the other studies linked to the antitumor effects of ceramide-based nano-formulations on melanoma cells (León et. al., 2006; Tran et al., 2008; Oskouian and Saba, 2010). Additionally, it was stated that the combinational treatment of nano-liposomal ceramide and sorafenib synergistically inhibited melanoma and breast cancer cell proliferation to reduce the development of the tumor (Tran et al., 2008). On the other hand, the effect of ATRA on the SM cycle was researched through sphingomyelinase (SMase) expression on MCF-7 (ATRA-sensitive) and MDA-MB-231 (ATRA-resistant) breast cancer cells (Tran et al., 2008). Accordingly, an increase in SMase activity was observed in the MCF-7 cell line, but not in the MDA-MB-231 cells, which are dependent on ATRA-sensitivity. In another study by Clarke et al. (2011), they reported that growth arrest in ATRA-induced MCF-7 breast cancer cells was mediated by the SMase expression. Estrogen receptor-positive MCF-7 cells were employed as a model system in order to investigate the involvement of nSMase 2 (nSMase2) and sphingolipids in the growth arrest produced by ATRA (Clarke et al., 2011). The findings indicated that ATRA leads to an elevation in ceramide levels and induces growth arrest in MCF-7 cells by upregulating the expression of nSMase2. Furthermore, nSMase2 was identified as the primary enzyme in the sphingolipid network of MCF-7 cells that was regulated by ATRA. These previous findings put the combinational approach forward as a prominent therapy for tumor-resistant agents such as ATRA.

With the implemented approach, the present study introduces the combinational therapy of ATRA and SM to synergistically improve activity and apoptosis, and hence attain a combinational anticancer effect on the in vitro B16F10 melanoma model, whereas determination of the safety of the treatment on immune cells was performed via cell viability studies on healthy macrophages. Also investigated was the overall effect of the combinational therapy on various cellular parameters, including cell viability, apoptotic cell death, and cell cycle, as well as gene expression levels to gain insights into the molecular interactions between the two active compounds and the B16F10 melanoma cells.

## 2. Materials and methods

### 2.1. Materials

The materials used in the study comprised 3-(4,5-dimethylthiazol-2-yl)-5-(3-carboxymethoxyphenyl)-2-(4-sulfophenyl)-2H-tetrazolium (MTS) assay (CellTiter96 AqueousOne Solution) (Promega UK Ltd., Chilworth, Southampton, UK); Annexin V-fluorescein isothiocyanate (FITC) Apoptosis Detection Kit (BD Biosciences Pharmingen, San Diego, CA, USA); propidium iodide and ribonuclease A (RNase A) (Sigma-Aldrich Chemie GmbH, Steinheim, Germany); octylphenoxypolyethoxyethanol (nonidet P-40) (AppliChem GmbH, Darmstadt, Germany); TRIzol reagent (Thermo Fisher Scientific Inc., Waltham, MA, USA); cDNA Synthesis Kit (Qiagen Inc., Germantown, MD, USA); and iTaq Universal SYBR Green Supermix (Bio-Rad, Hercules, CA, USA).

### 2.2. Cell culture

The murine macrophage RAW264.7 (TIB-71) and murine melanoma B16F10 (CRL-6475) cells were obtained from the American Type Culture Collection (Rockville, MD, USA). The cells were cultured in Dulbecco’s modified eagle’s high medium supplemented with 10% inactivated fetal bovine serum (Invitrogen Life Technologies, Waltham, MA, USA). The medium also contained 2 mM of L-glutamine, as well as 100 U/mL of penicillin and 100 μg/mL of streptomycin (Gibco, Thermo Fisher Scientific Inc.). The cells were maintained at 37 °C in a controlled atmosphere containing 5% CO_2_.

### 2.3. Cell viability analysis

The RAW264.7 macrophages and B16F10 melanoma cells were subjected to incubation in the presence of ATRA (in ethanol), SM, and various combinations of both for 24, 48, and 72 h. The determination of cell viability was conducted using MTS assay. Accordingly, the RAW264.7 and B16F10 cells were cultured in 96-well plates at a seeding density of 10,000 and 5,000 cells per well, respectively. Following incubation for 24, 48, and 72 h, the cells were subjected to treatment with varying doses of ATRA and SM at concentrations from 5 to 200 μM, both individually and in combination. After incubation, the cells were incubated in a solution of 10% MTS with glucose and 1X phosphate buffered saline (PBS) for 1 h. The absorbance at 490 nm was read using a microplate spectrophotometer (ELx800; BioTek Instruments Inc., Winooski, VT, USA). Cell viability was calculated as percentages by setting the absorbance of the untreated control group to 100%. The data were then analyzed using GraphPad Prism 8.0.1 (San Diego, CA, USA) to establish the half-maximal inhibitory concentration (IC_50_), and half-maximal cytotoxic concentration (CC_50_). The selectivity index (SI) of free ATRA and SM was calculated using the following equation: SI = CC_50_ in the RAW264.7 macrophage cell line / IC_50_ in the B16F10 melanoma cell line.

### 2.4. Cell cycle analysis by flow cytometry

The distribution of the cell cycle in the B16F10 cells was assessed by flow cytometry at 24 h. The B16F10 cells were seeded in 12-well plates with a density of 60,000 cells per well and incubated for 24 h. Following incubation, the cells were treated with ATRA (123 μM), SM (136 μM), and a combination of both (123 μM ATRA+136 μM SM) for 24 h. After 24 h of incubation and fixation with 70% ethanol, the cells were mixed with 0.01% nonidet P-40, and 100 μg/mL RNase A for 30 min. The analysis was performed with a flow cytometer for the gating of 10,000 cells after 5 min of incubation with 5 μg/mL of propidium iodide (PI). The percentages of cell populations were determined in the gap 0 (G0)/G1, synthesis (S), and G2/mitosis (M) phases.

### 2.5. Apoptosis assay

Apoptotic cell death of the B16F10 cells was analyzed via the Annexin V FITC/PI assay using a flow cytometer. Briefly, the B16F10 cells were seeded on 12-well plates with a density of 60,000 cells per well and incubated for 48 h. After incubation, the cells were treated with ATRA (123 μM), SM (136 μM), and a combination of both (123 μM ATRA+136 μM SM). After 48 h of incubation, they were subsequently exposed to Annexin V-FTIC for 15 min, followed by PI for 1 min, in line with the manufacturer’s instructions. The samples were examined using a FACSCalibur instrument (BD Biosciences, San Jose, CA, USA). A minimum of 10,000 cell counts were obtained for each data file. The gating procedure was appropriately adjusted to remove cellular debris, doublets, and clumps.

### 2.6. Quantitative PCR (qPCR) analysis

Primers for caspase 3 (CASP3) F: 5′ GGGAGCAAGTCAGTGGACTC 3′ and R: 5′ CCGTACCAGAGCGAGATGAC 3′; Bax F: 5′ TTGGAGCAGCCGCCCCAGG 3′ and R: 5′ CGGCCCCAGTTGAAGTTGCC 3′; programmed cell death-ligand 1 (PD-L1) F: 5′ TGGTCATTGTGCTGCTGCTA 3′ and R: 5′ TTACAGTTCGGCTGTCCACC 3′; cyclin-dependent kinase inhibitor 2A (CDKN2A) F: 5′ GAACTCGAGGAGAGCCATCTG 3′ and R: 5′ CCATCATCATCACCTGAATCGG 3′ were facilitated through the utilization of the Primer-BLAST online program, provided by the National Center for Biotechnology Information (Bethesda, MD, USA) and synthesized by Sentebiolab Biotech (Ankara, Turkey). The isolation of total RNAs from the samples was performed using TRIzol reagent. The cDNAs were synthesized using a cDNA-Synthesis Kit. iTaq Universal SYBR Green Supermix was employed in the qPCR analysis to measure the mRNA expression levels of the target genes. The gene ß-actin, a housekeeping gene, was employed to normalize the collected data. The iCycler reverse transcription (RT)-PCR machine (Bio-Rad) was utilized for all the RT-PCR assays.

### 2.7. Statistical analysis

GraphPad Prism Software (version 8.0.1) was employed for the statistical analyses. Error bars were utilized to visually represent the standard error of the mean. The datasets underwent initial ordinary one-way analysis of variance (ANOVA), followed by two-way ANOVA, and were subsequently scrutinized using Tukey’s multiple comparison test. Statistical significance was established at the following thresholds: * p ≤ 0.05, ** p ≤ 0.01, *** p ≤ 0.001, and **** p ≤ 0.0001.

## 3. Results

### 3.1. RAW264.7 macrophage and B16F10 melanoma cell viability after treatment with ATRA and SM

**Figure 1:**
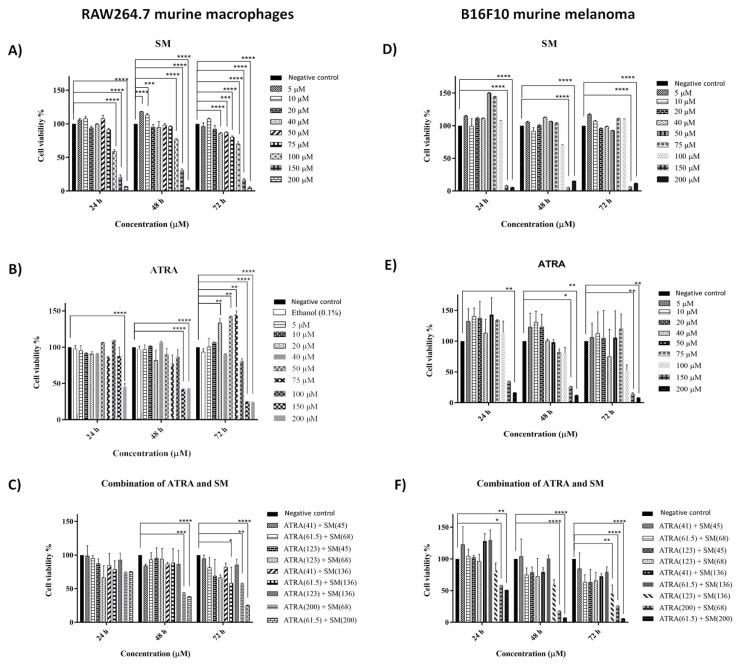
Cell viability analysis of the RAW264.7 macrophages (A, B, and C) and B16F10 melanoma cells (D, E, and F) treated with SM (A and D), ATRA (B and E), and their combination (C and F), respectively. Following treatment with various concentrations (5–200 μM) of free SM, free ATRA, and the combination of both, RAW264.7 and B16F10 cells were investigated in terms of cytotoxicity for 24, 48, and 72h. The data represent the mean of three independent experiments [(n = 3) ± SD].

The RAW264.7 macrophages and B16F10 melanoma cells were treated with ATRA, SM, and a combination of both at concentrations ranging between 5 and 200 μM for 24, 48, and 72h. In the combination treatments, the concentrations of ATRA (free) and SM (free) were based on their respective IC_50_ values, as follows: IC_50_/2 ATRA + IC_50_/2 SM, IC_50_/2 ATRA + IC_50_ SM, IC_50_ ATRA + IC_50_/2 SM, IC_50_/3 ATRA+ IC_50_/3 SM, IC_50_/3 ATRA + IC_50_ SM, IC_50_ ATRA + IC_50_/3 SM, 200 μM ATRA+ IC_50_/2 SM, IC_50_/2 ATRA+200 μM SM. In the positive control group, the cells were treated with 0.1% (v/v) ethanol to track the impact of the presence of the solvent (i.e. ethanol). The cell viability data of the MTS assay are presented in Figure 1.

The cell viability data of the RAW264.7 cells as a healthy cell line indicated that treatment with SM led to a gradual decrease in the viability of the RAW264.7 cells as the SM concentration increased to 150 μM (from 20% to 17%) and 200 μM (from 6% to 4%) (Figure 1A). On the contrary, there was no significant change in cell viability with 20 μM of SM (from 93% to 91%) at any of the time intervals. The viability of the RAW264.7 cells decreased insignificantly from 99% to 85% with 40 μM of SM, from 100% to 87% with 50 μM of SM, and from 91% to 80% with 75 μM of SM, within 24 and 72 h respectively. However, a significant decrease to 31% and 17% (p ≤ 0.0001) occurred when the RAW264.7 cells were treated with 150 μM of SM for 48 and 72 h, respectively (Figure 1A). On the other hand, cell viability decreased gradually to 25% and 22% with 150 and 200 μM of ATRA at 72 h, respectively (Figure 1B). No significant change occurred in the viability of the RAW264.7 macrophages when treated with 5, 10, and 40 μM of free ATRA at 24, 48, and 72 h.

Treatment with SM up to 100 μM caused no significant alteration in the viability of B16F10 melanoma cells, while treatment with 150 and 200 μM led to a significant decrease to 8% and 5% in the B16F10 cell viability (p ≤ 0.0001), respectively, at 24 h (Figure 1D). Similarly, up to a 15% decrease (p ≤ 0.0001) in cell viability was observed at 48 h with 200 μM of SM, whereas at 72 h, cell viability of the B16F10 cells was 6% and 11% (p ≤ 0.0001) with 150 and 200 μM of SM, respectively (Figure 1D). On the other hand, with 150 and 200 μM of ATRA, the cell viability decreased significantly below 15% at 72 h. With the 200 μM ATRA treatment, B16F10 cell viability decreased further down to 16%, 12%, and 8% at 24, 48, and 72 h, respectively (p ≤ 0.01) (Figure 1E). On the other hand, treatment with 5–50 μM of ATRA led to an increase in cell viability above 100% with low concentrations (i.e. 5–20 μM) at 24 and 48 h (Figure 1E).

The growth inhibition in the B16F10 and RAW264.7 cells allowed the calculation of both the IC_50_ and CC_50_ values for the free ATRA and SM (Figure S). While the IC_50_ values for the free ATRA and free SM were 123 ± 16.23 and 136 ± 15.03 μM at 72 h, respectively, for the B16F10 cells, the CC_50_ values for the free ATRA and SM were 99.54 ± 1.55 and 104.7 ± 2.05 μM, respectively, for the RAW264.7 macrophages. The selectivity index values for the ATRA and SM on the melanoma cancer model were 0.81 and 0.77, respectively. Considering the IC_50_ and CC_50_ values, among the effective concentrations on B16F10 melanoma cells, the optimum concentration of ATRA and SM for further combinational treatment studies (i.e. cell viability, apoptosis, cell cycle, and qPCR) was 123 and 136 μM, respectively, which was relatively safe in the healthy control RAW264.7 macrophage cells at 24, 48, and 72 h of incubation.

As shown in the cell viability analysis of the RAW264.7 cells after the combinational therapy (Figure 1C), there was no significant change in cell viability at 24 h with any of the concentrations, whereas 200 μM ATRA+68 μM SM and 61.5 μM ATRA+200 μM SM led to significant reductions in the macrophage cell viability at 48 and 72 h. Accordingly, the 200 μM ATRA+68 μM SM combination significantly decreased cell viability from 74% to 57% (p ≤ 0.0001) at 48 and 72 h. Likewise, the 61.5 μM ATRA+200 μM SM combination caused a significant decrease in cell viability to 25% at 72 h. On the contrary, the 123 μM ATRA+136 μM SM combination did not cause any significant change in the macrophage cell viability at 24, 48, and 72 h.

Figure 1F shows the potential anticancer effect of the SM and ATRA combinational therapy, which was attributed to increasing the biological effect of the compounds on the B16F10 cells with the improved safety properties in the RAW264.7 macrophages. Combinations of 61.5 μM ATRA+68 μM SM, 123 μM ATRA+68 μM SM, 41 μM ATRA+136 μM SM, and 123 μM ATRA+45 μM SM did not lead to any significant alterations in the B16F10 cell viability at 24 and 48 h. While combinational treatments with concentrations up to 123 μM ATRA and 136 μM SM did not cause a significant decrease in the cell viability at 24 and 48 h, the values indicated a gradual decrease in B16F10 cell viability compared to the individual molecular treatments in a time-dependent manner. Furthermore, 72 h of incubation led to a significant decrease to 45.74% compared to the untreated group (p ≤ 0.01). When the B16F10 cells were treated with the 200 μM ATRA+68 μM SM combination, there was a significant decrease in cell viability (i.e. 17.81% and 25.79%) at 48, and 72 h, respectively (p ≤ 0.0001). Similarly, the treatment of B16F10 melanoma cells with 61.5 μM ATRA+200 μM SM significantly decreased cell viability to 6.99% and 5.81% (p ≤ 0.0001) at 48 and 72 h, respectively (Figure 1F). Accordingly, these results revealed that the coadministration of 123 μM of ATRA and 136 μM of SM enhanced the anticancer efficacy in the B16F10 melanoma cells by mitigating their cytotoxic effects on the RAW264.7 macrophages.

### 3.2. Effects of ATRA and SM on B1610 cell morphology

**Figure 2:**
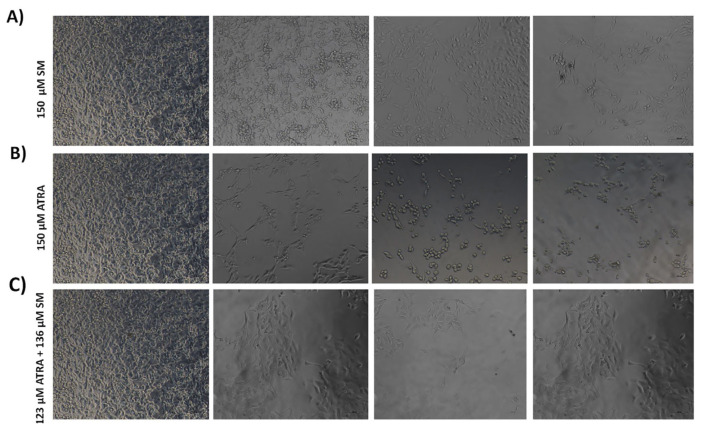
Phase-contrast microscopy images of the B16F10 cells treated with (A) 150 μM of SM, (B) 150 μM of ATRA, and (C) the combination of 123 μM of ATRA+136 μM of SM at 0, 24, 48 and 72 h. Bars correspond to 20 μm.

Phase-contrast microscopy was employed to observe alterations in the morphological characteristics of the B16F10 cells treated with a combination of ATRA and SM (Figure 2). The untreated cells exhibited a flattened adherence to the surface (Figure 2), whereas 150 μM of SM (Figure 2A), 150 μM of ATRA (Figure 2B), as well as the 123 μM ATRA+136 μM SM combination (Figure 2C), resulted in a significant alteration in the surface morphology and cell adhesion dramatically. The morphological characteristics of the flat surface underwent a transformation, resulting in a rounded spherical form, and they were separated from each other.

### 3.3. Cell cycle analysis

**Figure 3:**
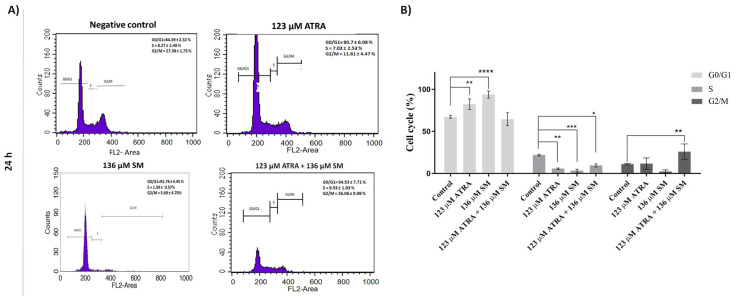
(A) Representative cell cycle histogram analysis and (B) cell cycle progression in the B16F10 cells following treatment with 123 μM of ATRA, 136 μM of SM, and the combination of both (123 μM of ATRA+136 μM of SM) for 24 h. The data represent the mean of three independent experiments [(n = 3) ± SD].

The cell cycle progression in the B16F10 cells after the SM and ATRA treatments was determined using flow cytometry analysis (Figure 3). The cell population percentage in the G0/G1 and G2/M phases varied depending on the treatment, demonstrating that cell cycle arrest was induced in the G0/G1 and G2/M phases at 24 h of incubation (Figure 3).

At the end of 24 h, the mean ± standard deviation of the G0/G1 phase for the control group was 64.39 ± 2.32%, whereas it was 80.70 ± 6.08% for 123 μM of ATRA, 92.76 ± 4.45% for 136 μM of SM, and 64.53 ± 7.71 for the 123 μM ATRA+136 μM SM combination (Figure 3A). Accordingly, as shown in Figure 3B, 123 μM of ATRA significantly increased the cell population (p ≤ 0.01) in the G0/G1 phase to 80.70 ± 6.08% in the B16F10 cells at 24 h. Similarly, the cell population in the G0/G1 phase increased significantly to 92.76 ± 4.45% with 136 μM of SM (p ≤ 0.0001), whereas the 123 μM ATRA+136 μM SM combination significantly increased the cell population to 34.06 ± 7.30% (p ≤ 0.01) in the G2/M phase at 24 h. Overall, these results indicated cell cycle arrest in the G2/M phases after the combinational treatment of ATRA and SM at 24 h.

### 3.4. Effects of ATRA and SM on apoptotic cell death

**Figure 4:**
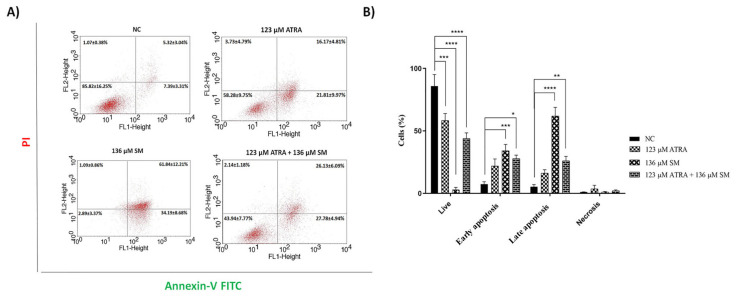
(A) Representative annexin-V FITC/PI histogram results and (B) a graph of the apoptotic cell death of the B16F10 cells following treatment with 123 μM of ATRA, 136 μM of SM, and the combination of both (123 μM of ATRA+136 μM of SM) for 48 h. The data represent the mean of three independent experiments [(n = 3) ± SD].

The apoptotic cell death in B16F10 cells following treatment with 123 μM of ATRA, 136 μM of SM, and the 123 μM ATRA+136 μM SM combination was assessed using Annexin V flow cytometry analysis, as illustrated in Figure 4, after 48 h. Accordingly, the apoptotic population fraction of the cells was 37.98% with 123 μM of ATRA, 96.03% with 136 μM of SM, and 53.91% with the 123 μM ATRA+136 μM SM combination at 48 h (Figure 4A). The early apoptotic population in B16F10 cells was 7.39% for the untreated control (NC), 21.81% for 123 μM ATRA, 34.19% for 136 μM SM, and 27.78% for the combined treatment. Meanwhile, the late apoptotic population rose from 5.32% in untreated cells to 16.17%, 61.84%, and 26.13% after exposure to 123 μM ATRA, 136 μM SM, and the combined treatment, respectively (Figure 4B). Furthermore, apoptotic cell death increased to 37.98% and 96.03% with 123 μM of ATRA and 136 μM of SM alone when compared to the untreated group (i.e. 12.71%), and the 123 μM ATRA+136 μM SM combination led to a significant increase to 53.91% at 48 h (p ≤ 0.001) (Figure 4B). On the contrary, the percentage of necrotic cells was negligible for all of the cell populations after incubation.

### 3.5. Effects of ATRA and SM on the gene expressions

**Figure 5:**
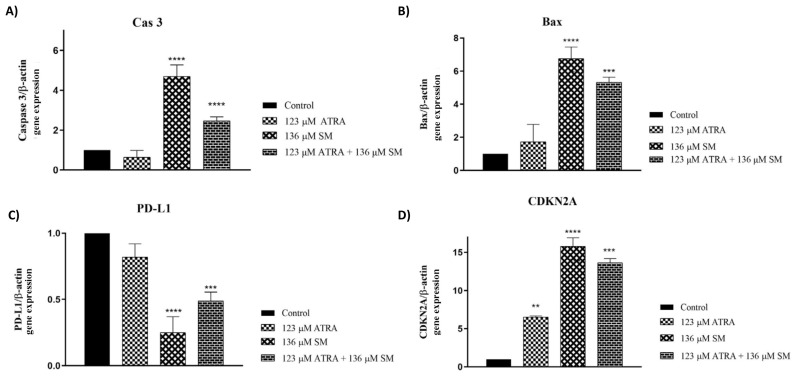
Fold increase of the relative (A) CASP3, (B) Bax, (C) PD-L1, and (D) CDKN2A expression using qPCR in B16F10 cells treated with 123 μM of ATRA, 136 μM of SM, alone, and the combination of both (123 μM of ATRA+136 μM of SM) at 24 h. The data represent the mean of three independent experiments [(n = 3) ± SD].

After treatment of the B16F10 cells with 123 μM of ATRA, 136 μM of SM, and the 123 μM ATRA+136 μM SM combination, the relative CASP3, Bax, PD-L1, and CDKN2A expressions of the cells were analyzed with qPCR at 24 h (Figure 5). The results showed that 136 μM of SM and the 123 μM ATRA+136 μM SM combination led to 5- and 2-fold increases in the CASP3 gene expression, respectively (p ≤ 0.0001) (Figure 5A). The B16F10 cells treated with 136 μM of SM significantly overexpressed the Bax gene by 6 times (p ≤ 0.0001), whereas the 123 μM ATRA+136 μM SM combination resulted in a 5-fold increase in the Bax gene expression (p ≤ 0.001) (Figure 5B). However, 123 μM of ATRA alone did not change the relative Bax gene expression (Figure 5B). On the other hand, the 123 μM ATRA+136 μM SM combination led to 0.13-fold (p ≤ 0.0001) and 0.5-fold (p ≤ 0.001) decreases in the PD-L1 gene expression in the B16F10 cells, respectively (Figure 5C). In addition, treatment of the B16F10 cells with 123 μM of ATRA, 136 μM of SM, and the 123 μM ATRA+136 μM combination resulted in 6-fold (p ≤ 0.01), 17-fold (p ≤ 0.0001), and 13-fold (p ≤ 0.001) increases in the CDKN2A gene expression, respectively, as depicted in Figure 5D.

## 4. Discussion

ATRA has been widely studied as a potent inhibitor of growth in melanoma cancer cells and displays very strong antineoplastic activity through the inhibition of cell proliferation, and induction of apoptosis in the cancer cells (Gallagher, 2002; Chen et al., 2019). Although ATRA indicates the efficacy in melanoma cells, it has some limitations, including the decrease in the concentrations due to its very short plasma pharmacokinetic profile, high volume of distribution, and excessive binding to tissues, thus leading to pharmacological limitations in its clinical use in cancer (Giuli et al., 2020). Similarly, SM has recently garnered much attention in the cancer investigations due to its potential anticancer effects as tumor-suppressing biologically active lipids (Huang et al., 2011; Ogretmen, 2017). However, the fast metabolic interconversions, and signaling functions occurring inside the biological membranes of sphingolipids pose biochemical and biophysical complexities when attempting to determine the precise roles of ceramides in the regulation of apoptosis. Furthermore, additional research is needed to investigate the potential modifications in sphingolipid signaling that could enhance immune cell (specifically macrophages) antitumor capabilities to improve the efficacy of immunotherapy in combating cancer. To address the limitations, a unique therapeutic approach involving the combination of ATRA and SM was devised herein for the treatment of melanoma, which has not been previously reported. The results demonstrated that the concomitant administration of ATRA and SM led to a substantial augmentation in anticancer activity, as evidenced by a notable decrease in the viability of the B16F10 melanoma cells with relatively less toxicity on the macrophages.

Cell viability data indicated that the combination of 123 μM of ATRA and 136 μM of SM significantly inhibited the cell proliferation of the B16F10 cells at 72 h in a time-dependent manner (Figure 1F). Furthermore, the combinational therapy improved the safety features of the ATRA and SM on the macrophages, in which the RAW264.7 viability was as high as 85.42% (Figure 1C) at 72 h of incubation with the 123 μM ATRA+136 μM SM combination compared to the standalone treatment of ATRA and SM (Figures 1A and 1B). At 48 h of incubation with 100 μM of ATRA, the viability of the melanoma cells was approximately 80%, indicating consistency with the value in the literature (i.e. 70%–75% and 85%) for MCF7 breast and HCT116 colon cancer cells, respectively (Carneiro et al., 2012). In addition, Bidad et al. (2011) reported that ≤10 μM of ATRA had no significant impact on cell viability and proliferation. The IC_50_ (123 ± 0.02 μM) and CC_50_ (99.54 ± 1.55 μM) values of the ATRA indicated a selectivity of 0.81 for the B16F10 cells compared to the RAW264.7 cells (Figure S). Previously, the effect of ATRA on the SM cycle was researched through SMase expression on MCF-7 (ATRA-sensitive) breast cancer cells, in which an increase in SMase activity was observed in the MCF-7 cell line in an ATRA-sensitivity-dependent manner (Tran et al., 2008). Clarke et al. (2011) reported that growth arrest in ATRA-induced MCF-7 breast cancer cells was mediated by SMase expression, and estrogen receptor-positive MCF-7 cells were employed as a model system to investigate the involvement of nSMase2 and sphingolipids in the growth arrest produced by ATRA. The underlying anticancer mechanism of the SM could be explained as the breakdown of SM by the SMase to produce ceramide after ATRA stimulation (Sheridan and Ogretmen, 2021). Moreover, ceramide can also be used as a substrate for the generation of complex sphingolipids via conversion to glucosylceramide by glucosylceramide synthase (Sheridan and Ogretmen, 2021). In terms of the anticancer mechanism, ceramide, a bioactive lipid, is thought to induce death, growth inhibition, and senescence in cancer cells, where classic mitochondria-dependent apoptosis was reported to be triggered by endogenous and exogenous ceramide signaling via ceramide-activated Ser-Thr protein phosphatases (CAPPs) and the expression of tumor suppressor genes (e.g., p38, Bax) (Liu et al., 2013). In addition, the effect of ceramide on CAPPs resulted in the inactivation of the antiapoptotic kinase AKT via protein dephosphorylation, and thus the downgrading of AKT by several signals (protein phosphatase 2A, protein kinase Cζ, and p38) decreases the phosphorylation of B-cell lymphoma 2 in intrinsic apoptosis (Zhou et al., 1998; Liu et al., 2013).

In line with the cell viability analysis, distinct morphological alterations were detected in terms of cell shrinkage, acquisition of a spherical form, and aggregation, as indicated by previous research (Saraste and Pulkki, 2000), suggesting the signs of apoptosis. Treatment with ATRA and SM resulted in the transformation of cells to round-shaped cell morphologies, which was attributed to the loss of integrity and detachment of the B16F10 cells induced apoptosis (Figure 2).

Parallel to the cell viability and morphological analysis, the cell cycle results suggested that the higher concentrations of ATRA and SM (i.e. ≥100 μM), alone, could alter the cell cycle frequencies at 24 h of treatment, thus inducing G0/G1 arrest in the B16F10 melanoma cells (Figure 3). The increase in the cell population in the G0/G1 phase and the induction of the cell cycle arrest by ATRA and ceramides alone in the cancer cells were compatible with the literature (Clarke et al., 2011; Liao et al., 2019). Similar to ATRA, another study stated that the addition of ceramide in human leukemic cell lines (e.g., MOLT-4) caused cell cycle arrest in the G0/G1 phase, and cell cycle progression, accompanied by an increase in ceramide levels resulting from the breakdown of SM (Jayadev et al., 1995). On the other hand, the contribution of ceramide in the G0/G1 arrest was closely linked to the role of nSMase2 in facilitating growth arrest triggered by confluence (Marchesini et al., 2004). Clarke et al. (2016) demonstrated that nSMase2, a key ceramide-producing enzyme involved in cellular stress responses, plays a role in ATRA-induced growth arrest induced by ATRA in MCF7 cells. The observed phenomenon was ascribed to the dephosphorylation of β-catenin through the involvement of a protein phosphatase 1-γ, indicating the presence of a signaling mechanism that mediates this particular action (Espaillat et al., 2015). Recent research has elucidated the role of dihydroceramide in regulating G0/G1 arrest triggered by cell confluence in neuroblastoma (Espaillat et al., 2015).

On the other hand, codelivery of ATRA with SM reduced the cell population in the G0/G1 phase in terms of G0/G1-phase cell cycle arrest, while leading to increased G2/M phase cell populations at 24 h (Figure 3). Previously, it was reported that ATRA treatment stimulates the conversion of SM into ceramides, an anticancer metabolite, by the induction of the SMase enzyme (Clarke et al., 2016; Ogretmen, 2017). Since ceramide induces apoptosis, cell differentiation, and senescence, the combination of SM and ATRA could be used as an anticancer agent by stimulating the ceramide accumulation mechanism. In addition, ceramide may have an involvement in regulating the G2/M cell cycle checkpoint (Espaillat et al., 2015). Previously, it was observed that the introduction of threo-1-phenyl-2-decanoylamino-3-morpholino-1-propanol, an inhibitor of glucosylceramide synthase, resulted in an increase in the levels of ceramide in NIH 3T3 cells (Espaillat et al., 2015). The increase in ceramide levels was attributed to a decrease in the cyclin-dependent kinase CDK1 activity, thus resulting in G2/M cell cycle arrest (Espaillat et al., 2015). The observed phenomenon in the current study can be ascribed to the arrest of the cell cycle in the G2/M phase following the combinational treatment of 123 μM ATRA+136 μM SM. Similarly, the modulation of G2/M arrest induced by the chemotherapeutic drug paclitaxel was observed by Espaillat et al. (2015), when β-glucosidase was knocked down, leading to the inhibition of ceramide formation (Espaillat et al., 2015). Elevation in ceramide levels has been documented to take place immediately during the G2/M transition in order to modulate the dephosphorylation of retinoblastoma (Rb) (Espaillat et al., 2015). Moreover, recent studies have indicated that ceramide could produce G2/M arrest in rhabdomyosarcoma cells.

The observation of elevated levels of CASP3 expression was consistent with the data obtained from the Annexin V FITC/PI flow cytometry analysis (Figure 4), indicating the incidence of higher cell death when the B16F10 cells were treated with the 123 μM ATRA+ 136 μM SM combination (Figure 5). Likewise, the CDKN2A gene was upregulated in the B16F10 melanoma cells when treated with 123 μM of ATRA, 136 μM of SM, and the 123 μM ATRA+136 μM SM combination, indicating consistency with the literature, in which germ-line mutations in the CDKN2A tumor-suppressor gene were related to the pathogenesis of hereditary melanoma, specifically concerning the development of melanoma. In addition, the gene CDKN2A is responsible for producing a cell-cycle regulator by suppressing the actions of CDK4 and CDK6, which are protein kinases that subsequently phosphorylate the Rb protein (Monzon et al., 1998). Herein, overexpression in the CDKN2A was correlated with apoptosis after the ATRA and SM combinational treatment in the melanoma cells, thus inducing tumor suppression and reduction in cell viability. On the other hand, the PD-L1 gene was downregulated in the B16F10 cells following treatment with 136 μM of SM and the 123 μM ATRA+136 μM SM combination (Figure 5C), verifying the results of previous studies in which ATRA exerted a suppressive effect on the expression of the PD-L1 gene in cellular systems (Tobin et al., 2018; Chen et al., 2019). Interestingly, 123 μM of ATRA alone did not lead to an alteration in the expression of the PD-L1 gene in B16F10 melanoma cells (Figure 5C). Recently, there have been effective applications of novel medicines that specifically target the surface expression of programmed cell death-1 (PD-1)/PD-L1 in the treatment of various solid malignancies, such as melanoma (Han et al., 2020). The expression of PD-L1 has been linked to the occurrence of tumor metastases, the advancement of tumors, and worse prognosis. The downregulation of the PD-L1 gene in the combinational treatments could be attributed to the increased tumor suppression and cell cycle arrest on melanoma cells. Overall, the findings of the cell cycle and apoptosis were supported by an investigation of the mRNA expression levels.

The combinational ATRA + SM treatment had a significant effect on cell proliferation and cell differentiation in melanoma cells, hence triggering apoptosis and causing cell cycle arrest. The combinational use of of 123 μM ATRA + 136 μM SM reduced cell viability, induced apoptotic cell death, caused cell cycle arrest, and overexpressed the Bax, CDKN2A, and CASP3 genes (Figures 1–5).

## 5. Conclusion

In summary, the hypothesis herein proposed that the concurrent use of two therapeutic components would augment the effectiveness of anticancer treatment in the treatment of B16F10 melanoma cancer cells, indicating the synergistic effects of this innovative treatment strategy. The findings provided evidence that the concurrent application of ATRA and SM results in a significant enhancement in anticancer efficacy, as indicated by a considerable reduction in the viability and proliferation of B16F10 melanoma cells with relative toxicity on RAW264.7 macrophages. The observed increase implies a possible interaction between the molecular pathways affected by ATRA and SM, resulting in a combined effect that exceeds the effects of each treatment on its own regarding the induction of apoptotic cell death, and cell cycle arrest on B16F10 cells.

The promising efficacy of the ATRA and SM combination in an in vitro model demonstrated remarkable efficacy, indicating its potential for future in vivo and clinical investigations in anticancer therapy. It is important to underscore that this study represents the first report revealing the combination of ATRA and SM enhancing the anticancer therapy on B16F10 melanoma cells.

## Supplementary Material

Figure S Graphical demonstrations of the 50% cytotoxic concentration (CC_50_) of (A) ATRA and (B) SM in the RAW264.7 macrophages.

## Footnotes

1 Information G, Melanoma A (2015). Melanoma Treatment (PDQ). PDQ Cancer Information Summaries [online]. Bethesda (MD): National Cancer Institute (US); 2002. Website https://www.ncbi.nlm.nih.gov/books/NBK66034/ [accessed 10 June 2024].
