# Supplementary figures and images for: Combinational therapy of all-trans retinoic acid (ATRA) and sphingomyelin induces apoptosis and cell cycle arrest in B16F10 melanoma cancer cells
Source: Turk J Biol. 2024 Oct 14;48(6):401–13. doi: 10.55730/1300-0152.2715 (PMC11698197; doi:10.55730/1300-0152.2715)

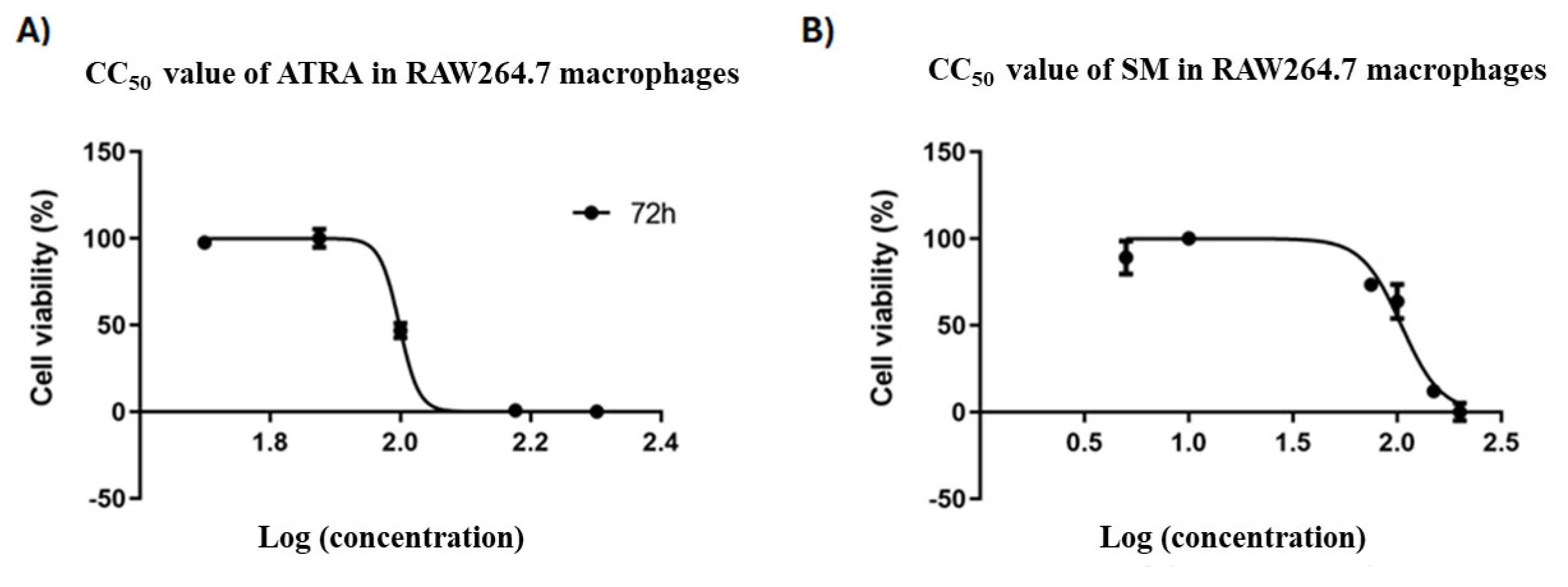

Supplement: Figure S — Graphical demonstrations of the 50% cytotoxic concentration (CC50) of (A) ATRA and (B) SM in the RAW264.7 macrophages. [file tjb-48-06-401s1.tif]
